# Blood Density Is Nearly Equal to Water Density: A Validation Study of the Gravimetric Method of Measuring Intraoperative Blood Loss

**DOI:** 10.1155/2015/152730

**Published:** 2015-01-29

**Authors:** Dominic J. Vitello, Richard M. Ripper, Michael R. Fettiplace, Guy L. Weinberg, Joseph M. Vitello

**Affiliations:** ^1^Department of Anesthesiology, Jesse Brown VA Medical Center, University of Illinois at Chicago, 820 S. Damen Avenue, Chicago, IL 60612, USA; ^2^Department of General Surgery, Jesse Brown VA Medical Center, University of Illinois at Chicago, 820 S. Damen Avenue, Chicago, IL 60612, USA

## Abstract

*Purpose*. The gravimetric method of weighing surgical sponges is used to quantify intraoperative blood loss. The dry mass minus the wet mass of the gauze equals the volume of blood lost. This method assumes that the density of blood is equivalent to water (1 gm/mL). This study's purpose was to validate the assumption that the density of blood is equivalent to water and to correlate density with hematocrit. *Methods*. 50 *µ*L of whole blood was weighed from eighteen rats. A distilled water control was weighed for each blood sample. The averages of the blood and water were compared utilizing a Student's unpaired, one-tailed *t*-test. The masses of the blood samples and the hematocrits were compared using a linear regression. *Results*. The average mass of the eighteen blood samples was 0.0489 g and that of the distilled water controls was 0.0492 g. The *t*-test showed *P* = 0.2269 and *R* ^2^ = 0.03154. The hematocrit values ranged from 24% to 48%. The linear regression *R* ^2^ value was 0.1767. *Conclusions*. The *R* ^2^ value comparing the blood and distilled water masses suggests high correlation between the two populations. Linear regression showed the hematocrit was not proportional to the mass of the blood. The study confirmed that the measured density of blood is similar to water.

## 1. Introduction

The quantity of intraoperative blood loss is often determined using subjective visual estimation by the operating surgeon or anesthesiologist. Several studies document that this visual estimation may underestimate actual blood loss by as much as 89% [1–5]. Some studies also report that as actual blood loss increases, estimated blood loss is increasingly inaccurate [6]. Accurately measuring blood loss during an operation can assist with fluid resuscitation and the need for transfusion. Overestimation of blood loss can lead to unnecessary transfusion practices. For research purposes, precise measurement of intraoperative blood loss is critical to compare different operative techniques or the effects of medications on blood loss.

During most operations, the direct measurement of the volume of blood collected in suction canisters is the most common clinical method used to determine intraoperative blood loss. When the volume of blood loss is small, the gravimetric method is a simple, accurate, and clinically relevant measurement technique [7]. With this method, surgical gauze sponges and laparotomy pads are weighed before and after use. The difference in their weight is generally believed to be an accurate measurement of blood loss. To convert the mass of blood to a more familiar volume statistic, knowledge of blood density is required. The density of blood is generally estimated to be one gram per milliliter [2, 7–9]. However, there is a paucity of documentation that the one milliliter of blood weighs one gram relationship is accurate or if it holds true for varying hematocrits. Existing data are conflicting and demonstrate blood density ranges between 1043 and 1060 kg/m^3^ (1.043–1.060 g/mL) [10]. The current study aims to determine the density of blood in an animal model and correlate it with the hematocrit. This will test the assumption that the specific gravity of blood is equal to water. If the relationship is valid and does not vary with hematocrit, it confirms the accuracy of weighing surgical sponges to determine intraoperative blood loss.

## 2. Materials and Methods

Rat blood used for this study was sampled from blood drawn as part of a protocol approved by the Animal Care Committee and Biologic Resources Laboratory at the University of Illinois at Chicago and the Institutional Animal Care and Utilization Committee of the Jesse Brown VA Medical Center (Chicago, Illinois). Male Sprague-Dawley rats, being utilized for another experiment and weighing between 385 and 435 grams, were sedated using atmospheric room air and 2% isoflurane. The rats were then placed on a heated surgical stage under a lightless heat lamp. The trachea was intubated and the rat ventilated with 1.7% isoflurane in oxygen. Cannulas were placed in both common carotid arteries and the left internal jugular vein. Heparinized microhematocrit tubes were filled with arterial whole blood from the cannulated left common carotid artery. The tubes were sealed at one end using two layers of white clay and centrifuged (International Equipment Company, Chattanooga, TN, Micro-Capillary Centrifuge, Model MB) at 10,000 revolutions per minute for 5 minutes. The hematocrit was read from the packed cell volume using a microcapillary reader (Damon/IEC Division Micro-Capillary Reader).

A few drops (<0.2 mL) of arterial whole blood obtained from the carotid artery were placed on a plastic weigh dish. A 50 *µ*L aliquot of this sample was collected using a disposable plastic micropipette (Fisher Scientific Fisherbrand, Loughborough, Leicestershire, United Kingdom, Finnpipette). This sample was placed on an electronic balance (Ohaus Corporation, Parsippany, NJ, Voyager) which had been previously zeroed, and the mass determined. Eighteen samples were collected and documented in this manner. Eighteen control trials using deionized water were also performed on the same electronic scale utilizing the identical technique as the blood samples. All measurements were determined at room temperature.

Statistical methods included a linear regression to provide a model for testing the correlation between blood density and hematocrit. Additionally, a Student's unpaired, one-tailed *t*-test with a Welch's correction was used to compare the means of the blood and distilled water masses. Data are reported as the mean ± SD.

## 3. Results and Discussion

**Figure 1:**
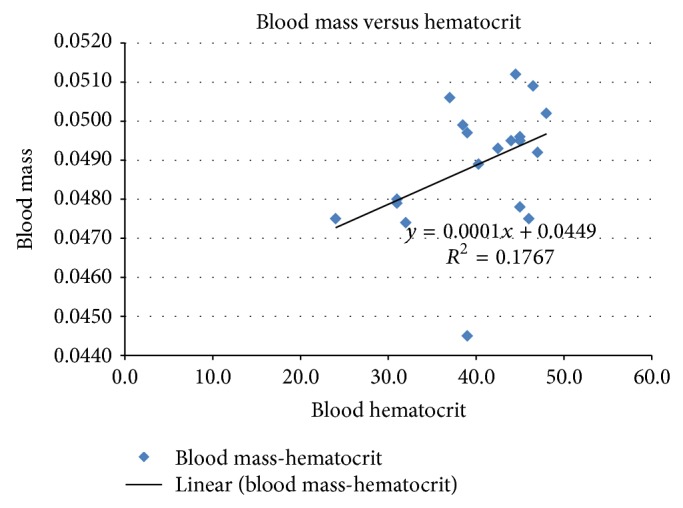
Graph correlating rat blood mass to blood hematocrit.

The masses of the 18 blood aliquots are recorded in Table 1. The average mass of the 50-microliter blood samples was 0.0489 ± 0.032 g with a range of 0.0445 g to 0.0512 g.  Table 2 lists the masses of the 18 distilled water controls. The average mass of the 50-microliter water samples was 0.0492 g with a range from 0.0487 g to 0.0496 g. The average density of the blood samples was normalized to the average mass of the distilled water controls by dividing the two means to yield specific gravity and an adjusted mean blood density of 0.994 ± 0.032 g/mL. The average hematocrit of the blood samples was 40.3% (range 24%–48%).  Figure 1 shows the mass of the 50 *µ*L samples of blood as a function of hematocrit. The *R* ^2^ value was 0.1767. Therefore, there was poor correlation between the hematocrits and the density of the blood samples. Populations were deemed fit for parametric testing but their variances were unequal by ANOVA (*P* < 0.00001). To account for this a Student's *t*-test with a Welch's correction was utilized to compare the blood and distilled water samples. The results yielded a *P* = 0.2269 and *R* ^2^ = 0.03154. This analysis verifies the fact that the mass of the blood and water samples was not significantly different.

The surgeon or anesthesiologist routinely estimates the volume of blood lost during each operation. This estimate can guide transfusion requirements or assist in the amount of intravenous resuscitation required to maintain euvolemia. Accurate measurement of intraoperative blood loss also allows the comparison of different surgical techniques and methods to minimize blood loss during surgery. Inaccurate determination of intraoperative blood loss can lead to unnecessary transfusions. There is ample evidence to suggest that blood is immunosuppressive [11, 12] and has been associated with worse outcomes in colorectal cancer [13]. Blood transfusion also increases the risk of surgical site infections [14] and is expensive.

There are many methods to determine the volume of intraoperative blood loss. The most precise method utilizes spectrophotometry [15–17]. This method prepares a referenced standard of the patient's own blood. Blood lost into drapes, gowns, and sponges is extracted and filtered. The resulting sample is then read in a spectrophotometer along with the patient's reference standard. This method is considered highly reliable; however, it is expensive, labor intensive, and clinically impractical.

During a typical operation, shed blood is aspirated via a suction system directly into canisters where the blood volume can be accurately measured. Blood lost into sponges and laparotomy pads is also visually estimated by the attending surgeon or anesthesiologist—a routine but subjective and highly inaccurate method [1–5]. The gravimetric method of determining blood loss requires weighing surgical sponges before and after use. The difference in weight is assumed to be the volume of blood lost as measured in milliliters. This assessment is based upon density which is defined as mass per unit volume. Water density is dependent upon temperature. At room temperature of 23°Centigrade, water has a density of 0.997538 g/mL [12]. This translates into the common metric equivalent that 1 gram of water is equal to 1 milliliter. The *P* value of 0.2269 for this study demonstrates that the two populations are not statistically significantly different. The *R* ^2^ for the two populations was 0.03154. This low value demonstrates that the means of the water and the blood samples are statistically similar. The relationship of the mass of blood and the hematocrit has not been clearly established [13, 14]. Our study confirmed that there was poor correlation between the mass of blood and hematocrits varying between 24 and 48 percent (Figure 1).

The purpose of this study was to validate the commonly held assumption that the density of blood is one gram per milliliter. We found the average density of blood was 0.994 g/mL ± 0.032 g. Notably, changes in the hematocrit did not affect the density of the blood samples and analysis revealed no relationship between the two parameters. These data support the belief that the density of blood and water is very close and therefore the act of weighing gauze sponges before and after use in the operating room is a reliable guide to determine the volume of blood lost into the gauze.

There were some limitations to this study. A 50 *µ*L sample of water should have a mass of 0.05 grams although none of the samples were recorded with this value and probably reflects the error of pipetting and weighing. Aliquots were recorded immediately after dispensing. This minimized any errors due to evaporation. It was also noted that the blood samples adhered to the inside of the micropipette tip, possibly causing the dispensed volume to be less than actually measured. The quantity of blood or water retained on the inside of pipettes after dispensing the fluid was unknown and not measured, but presumably constant. Additional considerations included the fact that the density of water is a function of temperature, nearing a peak value of 999.9720 kg/m^3^ at 4°C [18]. At a temperature of 23°C, water has a density of 0.997538 g/mL [14] which offers a potential explanation for the control group's deviation from an expected density of 1 gm/mL. More sophisticated methods to determine mass could be used to reproduce and validate the results. A greater sample size may help to confirm the accuracy of the findings and repeating the study across a greater range of hematocrits would provide additional useful information. Finally, a direct comparison of rat and human blood densities is lacking in the literature. Based upon comparisons of other mammalian species, [19, 20] however, it is suggested that the comparative densities between human and rat blood are probably valid.

## 4. Conclusion

The measured mass of blood is nearly equal to distilled water. This confirms the assumption that the densities of blood and of distilled water are nearly equivalent. This helps verify the use of the gravimetric method of weighing sponges in the operating room to accurately determine blood loss and a one-gram increase in the weight of a blood soaked surgical gauze is equal to one milliliter of blood lost.

## Figures and Tables

**Table 1:** Masses of the eighteen rat blood samples.

50 *μ*L samples of blood		
Number	Hematocrit	Mass (g)
1	45.0	0.0496
2	31.0	0.0479
3	44.5	0.0512
4	37.0	0.0506
5	48.0	0.0502
6	38.5	0.0499
7	46.5	0.0509
8	39.0	0.0497
9	47.0	0.0492
10	45.0	0.0478
11	42.5	0.0493
12	32.0	0.0474
13	46.0	0.0475
14	39.0	0.0445
15	45.0	0.0495
16	31.0	0.0480
17	24.0	0.0475
18	44.0	0.0495

Average	40.3	0.0489
Max	48.0	0.0512
Min	24.0	0.0445
Range	24.0	0.0067
SD	6.9	0.0016

**Table 2:** Masses of the eighteen deionized water controls.

50 *μ*L deionized water controls	
Control number	Mass (g)
1	0.0495
2	0.0495
3	0.0494
4	0.0496
5	0.0496
6	0.0487
7	0.0494
8	0.0495
9	0.0490
10	0.0489
11	0.0491
12	0.0492
13	0.0489
14	0.0490
15	0.0492
16	0.0491
17	0.0493
18	0.0487

Average	0.0492
Max	0.0496
Min	0.0487
Range	0.0009
SD	0.0003
